# Pleasantness Ratings for Harmonic Intervals With Acoustic and Electric Hearing in Unilaterally Deaf Cochlear Implant Patients

**DOI:** 10.3389/fnins.2019.00922

**Published:** 2019-09-03

**Authors:** Emily R. Spitzer, David M. Landsberger, David R. Friedmann, John J. Galvin

**Affiliations:** ^1^Department of Otolaryngology–Head and Neck Surgery, New York University School of Medicine, New York, NY, United States; ^2^House Ear Institute, Los Angeles, CA, United States

**Keywords:** cochlear implant, music perception, single-sided deafness, dissonance, harmonic intervals

## Abstract

**Background:**

Harmony is an important part of tonal music that conveys context, form and emotion. Two notes sounded simultaneously form a harmonic interval. In normal-hearing (NH) listeners, some harmonic intervals (e.g., minor 2nd, tritone, major 7th) typically sound more dissonant than others (e.g., octave, major 3rd, 4th). Because of the limited spectro-temporal resolution afforded by cochlear implants (CIs), music perception is generally poor. However, CI users may still be sensitive to relative dissonance across intervals. In this study, dissonance ratings for harmonic intervals were measured in 11 unilaterally deaf CI patients, in whom ratings from the CI could be compared to those from the normal ear.

**Methods:**

Stimuli consisted of pairs of equal amplitude MIDI piano tones. Intervals spanned a range of two octaves relative to two root notes (F3 or C4). Dissonance was assessed in terms of subjective pleasantness ratings for intervals presented to the NH ear alone, the CI ear alone, and both ears together (NH + CI). Ratings were collected for both root notes for within- and across-octave intervals (1–12 and 13–24 semitones). Participants rated the pleasantness of each interval by clicking on a line anchored with “least pleasant” and “most pleasant.” A follow-up experiment repeated the task with a smaller stimulus set.

**Results:**

With NH-only listening, within-octave intervals minor 2nd, major 2nd, and major 7th were rated least pleasant; major 3rd, 5th, and octave were rated most pleasant. Across-octave counterparts were similarly rated. With CI-only listening, ratings were consistently lower and showed a reduced range. Mean ratings were highly correlated between NH-only and CI-only listening (*r* = 0.845, *p* < 0.001). Ratings were similar between NH-only and NH + CI listening, with no significant binaural enhancement/interference. The follow-up tests showed that ratings were reliable for the least and most pleasant intervals.

**Discussion:**

Although pleasantness ratings were less differentiated for the CI ear than the NH ear, there were similarities between the two listening modes. Given the lack of spectro-temporal detail needed for harmonicity-based distinctions, temporal envelope interactions (within and across channels) associated with a perception of roughness may contribute to dissonance perception for harmonic intervals with CI-only listening.

## Introduction

Along with language, music is a near-universal part of the human experience. Although cochlear implants (CIs) can enable those with severe hearing loss to understand speech with high levels of intelligibility, these devices are extremely poor at conveying most tonal aspects of music (e.g., melody and harmony) that are crucial for music perception and appreciation. The coarse spectro-temporal resolution provided by a CI is adequate for speech recognition, due to the availability of low-frequency temporal envelope cues. However, this coarse resolution is not sufficient for music perception, especially perception of pitch, timbre, harmonicity, etc. (e.g., Smith et al., 2002; Shannon et al., 2004). The limited number of implanted electrodes, the broad acoustic-to-electric frequency allocation, the strong channel interaction among the implanted electrodes, and patient-related idiosyncrasies in terms of the position of the electrodes in relation to healthy neural populations (the “electrode-neural interface”) all contribute to distorted perception of musical intervals, relative to acoustic hearing (e.g., McDermott, 2004; Galvin et al., 2007, 2009a; Gfeller et al., 2007; Nimmons et al., 2008; Kang et al., 2009; Cousineau et al., 2010; Limb and Roy, 2014). Distorted perception of melodic (i.e., sequential) intervals contributes to CI users’ poor melody perception, especially when rhythm cues are unavailable (e.g., Gfeller et al., 2002, 2007; Kong et al., 2004; Vongpaisal et al., 2006). With polyphonic music (multiple instruments or voices), CI users’ melodic pitch perception worsens further (e.g., Galvin et al., 2009b, 2012; Penninger et al., 2013, 2014; Crew et al., 2015). While perception of melodic intervals has been extensively studied, relatively little is known about CI users’ perception of harmonic (i.e., simultaneous) intervals.

Simultaneous presentation of musical intervals forms the basis of harmony and is used to build musical chords. Depending on the component notes, harmonic intervals may be perceived as having different degrees of consonance or dissonance, sounding complementary, pleasant, unpleasant or neutral (e.g., Dowling and Harwood, 1986; Deutsch, 2007; Bidelman and Krishnan, 2009). With normal hearing (NH), some harmonic intervals may sound harsh or dissonant (e.g., minor 2nd, tritone), while others may sound more pleasing (e.g., major 3rd, 5th). When notes are combined, the harmonic spectra and temporal properties of each component note are also combined. As such, the degree of “harmonicity” in the combined spectrum (which relates to the spacing of the harmonics) may contribute to perceived consonance (e.g., Tramo et al., 2001; McDermott et al., 2010). Temporal beating or roughness may also contribute to perceived dissonance for harmonic intervals (e.g., Plomp and Levelt, 1965; Tramo et al., 2001). Individual preferences for consonance may also be influenced by musical training (McDermott et al., 2010) and experience with Western musical structure (McDermott et al., 2016). Perception of dissonance contributes strongly to emotional responses to music (Fritz et al., 2009), as does the perception of harmonic “syntax” (how harmonic intervals and chords relate to each other in a piece of music; e.g., Patel, 2003).

It is unclear whether CI users are able to perceive dissonance for harmonic intervals with electric hearing. Brockmeier et al. (2011) reported that CI users were able to discriminate musical chords, but that discrimination was poorer than that of NH listeners. Caldwell et al. (2016) altered the accompanying chords to a melody to be overtly consonant or dissonant; pleasantness ratings decreased as the degree of dissonance in the accompaniment increased for NH listeners, but not for CI listeners. Knobloch et al. (2018) reported that CI users were able to discriminate chords, and that chord preference ratings (from top to bottom: major, minor, suspended 4th, augmented, diminished, and diminished 5th) were generally similar to those of NH listeners. These studies suggest that some differentiation among harmonic intervals is possible with electric hearing, but perception of dissonance for specific intervals has yet to be reported.

Arguably, the greatest benefit for CI users’ music perception has been the inclusion of acoustic hearing (where possible) in the ear contralateral or ipsilateral to the CI ear. CI indications have expanded to allow for increasing amounts of acoustic hearing in the contralateral ear. Preservation of residual low-frequency acoustic hearing in the implanted ear is becoming more frequent due to advanced electrode designs and surgical techniques (Santa Maria et al., 2014; Causon et al., 2015; Wanna et al., 2015; Sierra et al., 2019). Many studies have shown significant benefits for music perception with combined acoustic and electric hearing over CI-only performance (e.g., Kong et al., 2005; Dorman et al., 2008; Vermeire et al., 2008; Sucher and McDermott, 2009; Cullington and Zeng, 2011; Crew et al., 2015; Cheng et al., 2018). Depending on the listening task, binaural enhancement over acoustic hearing may be limited. For example, Crew et al. (2015, 2016) found no significant binaural enhancement over acoustic hearing alone for melodic contour identification; some participants even exhibited binaural interference relative to acoustic hearing alone, suggesting that acoustic and electric stimulation patterns were not optimally combined. The data also suggested that for melodic pitch perception, the CI contributed very little to bimodal perception, due to the availability of temporal fine-structure (TFS) cues with acoustic hearing that are important for melodic pitch perception.

Over the last decade, increasing numbers of patients with single-sided deafness (SSD) have undergone cochlear implantation. SSD-CI patients have normal or near-normal acoustic hearing in one ear and a CI in the other ear. Many studies have shown significant benefits of cochlear implantation for SSD patients in terms of reduced tinnitus severity, as well as improved localization, speech understanding and quality of life (e.g., Van de Heyning et al., 2008; Vermeire et al., 2008; Vermeire and Van de Heyning, 2009; Firszt et al., 2012; Mertens et al., 2015; Friedmann et al., 2016; Arndt et al., 2017; Dillon et al., 2017a, b; Galvin et al., 2018). Landsberger et al. (2019) reported significantly higher musical sound quality ratings when SSD-CI participants listened with acoustic and electric hearing than with acoustic hearing alone. SSD-CI patients represent a unique patient population with which to compare auditory perception between acoustic and electric hearing within participants [e.g., inter-aural pitch matching (Vermeire et al., 2008, 2015; Goupell et al., 2019); sound quality differences between acoustic and electric hearing (Vermeire et al., 2013; Dorman et al., 2017); melodic interval distortion (Todd et al., 2017)]. SSD-CI patients would be similarly valuable for comparing harmonic interval perception between acoustic and electric hearing. Pleasantness ratings for different intervals obtained with acoustic hearing can provide an accurate reference for ratings obtained with electric hearing in the same listener (rather than across NH and CI listeners, as in most previous studies).

In the present study, pleasantness ratings were obtained for harmonic intervals in SSD-CI participants with the NH ear alone, the CI ear alone, and with both ears. We expected that ratings with the NH ear would be similar to those found for NH listeners in previous studies (McDermott et al., 2010; Cousineau et al., 2012). However, we hypothesized that due to the poor spectro-temporal resolution with the CI (which would not support harmonicity cues that are important to consonance perception), pleasantness ratings would be generally poor with electric hearing and would not correspond to ratings with acoustic hearing. Despite the expected poor ratings with the CI, we hypothesized significant binaural enhancement over the NH ear alone, similar to Landsberger et al. (2019) and in agreement with anecdotal reports from SSD-CI patients that music sounds better with combined acoustic and electric hearing than with acoustic hearing alone.

## Experiment 1: Harmonic Interval Perception

### Methods

#### Participants

Eleven SSD-CI patients (5 males, 6 females) participated in the study. All participants were adults with post-lingual SSD, with profound hearing loss in one ear and normal or near-normal hearing in the contralateral ear. Six participants used Cochlear Ltd. Devices (codes begin with “N”), four used MED-EL devices (codes begin with “M”), and one used an Advanced Bionics (AB) device (code begins with “C”). Two participants (N10 and C1) reported extensive formal music education, one reported informal musical education (N6), and the other eight reported no music training. Average age was 53.91 years (range: 27–70 years). Further demographic information can be found in Table 1. All participants were paid for their participation and provided written informed consent in accordance with the Institutional Review Board Procedures of New York University (IRB #S14-00809 and #S14-00435) and in accordance with the Declaration of Helsinki.

**TABLE 1:** Demographic information for SSD-CI subjects.

**Participant**	**Lab code**	**Dur deaf (yrs)**	**CI exp (yrs)**	**Etiology**	**Device**	**Strategy**	**CI ear**	**Non-CI ear PTA (dB HL)**	**CI ear CNC % correct**
C1	SSD-C1	2.6	3.01	Idiopathic SSHL	AB HiRes90k Mid Scala	HiRes Optima-P	R	8.3	30
M2	SSD-M1	0.3	1	Cochlear Schwannoma/NF2	MED-EL Synchrony Flex 28	FS4-P	R	25.0	4
M3	SSD-M2	1.3	0.31	Idiopathic progressive	MED-EL Synchrony Flex 28	FS4-P	L	11.7	30
M4	SSD-M3	0.9	2.45	Idiopathic SSHL	MED-EL Synchrony Flex 28	FS4-P	L	26.7	44
M5	SSD-M4	6.6	1.07	Idiopathic SSHL	MED-EL Synchrony Flex 28	FS4-P	L	5.0	DNT
N6	SSD-N1	4.2	8.26	Idiopathic SSHL	Cochlear N512 CI512	ACE	R	43.3	70
N7	SSD-N6	2.4	3.3	Idiopathic SSHL	Cochlear Profile CI512	ACE	R	3.3	84
N8	SSD-N7	0.4	4.21	Idiopathic SSHL	Cochlear Profile CI512	ACE	R	15.0	48
N9	SSD-N8	1.1	1.56	Genetic	Cochlear Profile CI532	ACE	L	8.3	92
N10	SSD-N9	9	0.79	Temporal bone fracture	Cochlear Profile CI512	ACE	L	16.7	66
N11	SSD-N10	3.2	1.95	Idiopathic SSHL	Cochlear Profile CI532	ACE	R	26.7	70

*For simplicity, subjects are referred to in this manuscript by subject code (i.e., C1-N11). The letter before the number refers to the manufacturer of the device (“C”: Advanced Bionics; “M”: MED-EL, and “N”: Cochlear). The internal lab code for each subject is also provided for comparison purposes to other publications. Dur deaf = duration of deafness. CI exp = cochlear implant experience. AB = Advanced Bionics. ACE = Advanced Combination Encoder. PTA = pure-tone average thresholds across 500, 1000, and 2000 Hz. DNT = did not test. For all subjects except for C1, M3, and M5, CNC scores with the CI-only were obtained after 1 year of CI experience. For C1 and M3, scores were obtained after 3 months of CI experience; no scores were available for M5.*

#### Stimuli

Stimuli were equally tempered MIDI piano notes generated using Matlab and a MIDI library by Ken Schutte^1^. Each note was 1s in duration. For a given interval, both notes were normalized to have the same root-mean-square (RMS) amplitude (−26.9 dBFS) and then mixed together. Stimuli were ramped on and off to eliminate sharp onsets and offsets. All samples were recorded with a 44.1 kHz sampling rate at 16-bit depth. Harmonic interval stimuli are shown in Table 2. Each interval was comprised of one of two “root notes” [F3 (≈175 Hz) or C4 (≈262 Hz)] presented simultaneously with a second note. In this study, “root note” is defined as the lower note in the interval. It was important to test two different root notes to avoid entrainment to a particular frequency, and to accommodate potential differences across SSD-CI patients in terms of frequency allocation and the electrode-neural interface. Given these potential differences, ratings might differ across root notes for particular subjects. Two “interval spans” were also tested: 1–12 semitones (“within-octave”) and 13–24 semitones (“across-octave”). For NH listeners, there is some evidence that harmonic intervals separated by an octave or more are perceived to be more consonant than the same interval presented within an octave (Plomp and Levelt, 1965; Kameoka and Kuriyagawa, 1969). While it was unclear if this relationship would hold for CI listeners, we hypothesized that many of the within-octave intervals would be represented on the same channel (due to the clinical CI frequency allocation). This would result in some amount of within-channel temporal beating. Even for larger within-octave intervals, channel interactions may cause both notes to be represented by overlapping neural populations. Including a set of intervals presented across-octave allows for component notes to be delivered to different electrode locations and increases the likelihood that notes will be encoded by separate neural populations. Thus, pleasantness ratings for a given interval could be compared within or across channels. The interval span conditions may also shed light on the importance of place cues for dissonance perception. Table 2 shows the within- and across-octave intervals. Overall, there were 24 stimuli per root note, for a total of 48 test intervals.

**TABLE 2:** Test intervals for Exp. 1.

**Within-octave**	**Interval:**		**Minor 2nd**	**Major 2nd**	**Minor 3rd**	**Major 3rd**	**4th**	**Tritone**	**5th**	**Minor 6th**	**Major 6th**	**Minor 7th**	**Major 7th**	**Octave**
	
		**Semitones:**	**1**	**2**	**3**	**4**	**5**	**6**	**7**	**8**	**9**	**10**	**11**	**12**
	F3	Note: F3 +	F#3	G3	G#3	A3	A#3	B3	C4	C#4	D4	D#4	E4	F4
		MIDI note: 53 +	54	55	56	57	58	59	60	61	62	63	64	65
		Frequency: 175 Hz +	185	196	208	220	233	247	262	277	294	311	330	349
	C4	Note: C4 +	C#4	D4	D#4	E4	F4	F#4	G4	G#4	A4	A#4	B4	C5
		MIDI note: 60 +	61	62	63	64	65	66	67	68	69	70	71	72
		Frequency: 262 Hz +	277	294	311	330	349	370	392	415	440	466	494	523

**Across-octave**	**Interval:**		**Minor 9th**	**Major 9th**	**Minor 10th**	**Major 10th**	**11th**	**Diminished 12th**	**12th**	**Minor 13th**	**Major 13th**	**Minor 14th**	**Major 14th**	**Double Octave**
	
		**Semitones:**	**13**	**14**	**15**	**16**	**17**	**18**	**19**	**20**	**21**	**22**	**23**	**24**

	F3	Note: F3 +	F#4	G4	G#4	A4	A#4	B4	C5	C#5	D5	D#5	E5	F5
		MIDI note: 53 +	66	67	68	69	70	71	72	73	74	75	76	77
		Frequency: 175 Hz +	370	392	415	440	466	494	523	554	587	622	659	698
	C4	Note: C4 +	C#5	D5	D#5	E5	F5	F#5	G5	G#5	A5	A#5	B5	C6
		MIDI note: 60 +	73	74	75	76	77	78	79	80	81	82	83	84
		Frequency: 262 Hz +	554	587	622	659	698	740	784	831	880	932	988	1047

*The top section shows within-octave intervals (1–12 semitones) and the bottom section shows across-octave intervals (13–24 semitones). Two root notes (F3 and C4) were tested for each interval; the different rows show the notes in terms of musical notation, MIDI notation, and frequency. The columns show the name, symbol, and semitone distance from the root note.*

Stimuli were presented using custom software via audio device (Tascam US-322), and were routed to the NH ear via circumaural headphone (Sony MDR-7506) and to the CI ear via isolated direct audio input (DAI). Three listening conditions were tested: NH-only (monaural), CI-only (monaural), and NH + CI (diotic binaural). For MED-EL users, the “red” DAI cable was connected between the audio device and the CI processor, which provided a mix of 90% audio input and 10% microphone input. Participants were tested with their clinical settings. For Cochlear users, participants’ clinical maps were programed onto loaner N6 (CP910) processors configured for DAI input only. Similarly, for the AB user, the clinical map was programed onto a loaner Harmony processor; the map was configured for DAI input only.

#### Procedure

Before testing began, all participants were asked to set the loudness of the stimuli presented to the NH and CI ear to an equally loud most-comfortable level. A musical interval was played alternately between the CI ear and the NH ear, and the participant adjusted the output volume of each channel of the audio device until both channels were equally loud and at the most-comfortable listening level. After these initial adjustments, participants were not allowed to adjust the volume of either output for the remainder of the experiment.

All three listening conditions and all 48 intervals were presented within each test block (144 stimuli in total). Listening conditions and intervals were randomized within each test block. A total of five blocks (i.e., five repetitions of each interval) were tested for each participant; the test blocks were administered during a single session lasting approximately 3 hours. During testing, a stimulus was randomly selected from the stimulus set and presented to the participant, who was asked to rate the pleasantness by clicking on a continuous bar with the anchors “least pleasant” and “most pleasant” at either end (no other scaling of the range was provided). A short practice session was given prior to starting the experiment to familiarize participants with the stimuli and test procedure. Participants were allowed to repeat each stimulus as many times as they wanted. Pleasantness ratings were averaged for each interval, root note, and listening conditions across the five repetitions from each test block.

### Results

**FIGURE 1:**
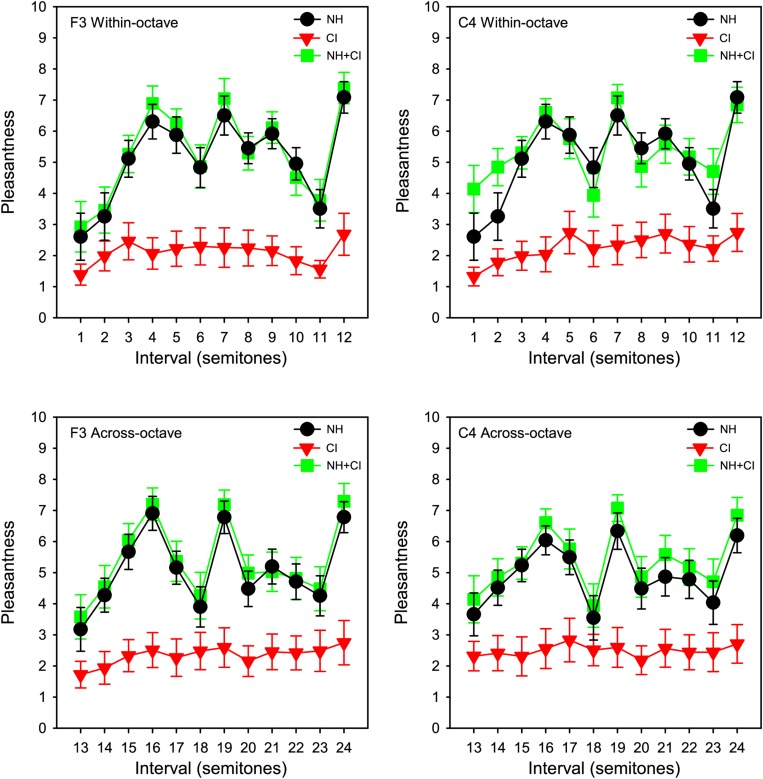
Mean pleasantness ratings for SSD-CI participants (*n* = 11) with NH-only (black), CI-only (red), and NH + CI (green) listening as a function of interval size. Interval size in semitones is shown on the abscissa. Data for the F3 and C4 root notes are shown in the right and left columns, respectively. Data for within- and across-octave intervals are shown in the top and bottom rows, respectively. The error bars show the standard error.

Pleasantness ratings were scaled from 0 to 10 according to where participants clicked on the rating bar. Figure 1 shows group (*n* = 11) mean pleasantness ratings for the NH-only, CI-only and NH + CI listening conditions as a function of interval size. Consistent with McDermott et al. (2010), for NH-only listening, some intervals were consistently rated least pleasant (minor 2nd, tritone, and major 7th), while others were consistently rated most pleasant (major 3rd, 5th, and octave). Ratings with CI-only listening were much poorer than with NH-only or NH + CI listening. Ratings were similar between NH-only and NH + CI listening. A four-way repeated measures analysis of variance (RM ANOVA) was performed with listening condition (NH, CI, NH + CI), interval span (within-octave, across-octave), root note (F3, C4) and interval size (12 levels: 1–12 semitones) as within-subject factors. Significant main effects were observed only for listening condition [*F*(1,10) = 18.115, *p* < 0.001] and interval size [*F*(11,110) = 14.95, *p* < 0.001]. A summary of the analysis is shown in Supplementary Appendix 1. We also analyzed the data using a 3-way RM ANOVA, removing the factor of interval span and thus considering intervals from 1 to 24 semitones. Results were nearly identical to those from the above the 4-way RM ANOVA, with the exception of an interaction between listening condition, interval size, and root note [*F*(46, 460) = 1.48, *p* = 0.027].

*Post hoc* analyses were conducted to compare the NH-only and NH + CI listening conditions. To observe whether there was any binaural enhancement for individual participants, paired *t*-tests were conducted on individual participants’ NH-only and NH + CI data. Because the four-way RM ANOVA showed no significant effect of interval span or root note, data were combined for a total of 48 comparisons between the NH-only and NH + CI listening conditions within each participant. Complete results are shown in Supplementary Appendix 2. Results showed no significant difference between NH-only and NH + CI ratings for 7 of the 11 participants (*p* > 0.05 in all cases). For two participants, there was significant binaural enhancement [N7: *t*(47) = −30.96, *p* < 0.001; N9: *t*(47) = −19.54, *p* < 0.001]. For another two participants, there was significant binaural interference [C1: *t*(47) = 2.54, *p* = 0.015; M2: *t*(47) = 13.91, *p* < 0.001]. Pearson correlation analysis showed no significant relationship between the degree of binaural enhancement (NH + CI – NH-only) and the difference between NH and CI ratings (*r* = −0.04, *p* = 0.901).

**FIGURE 2:**
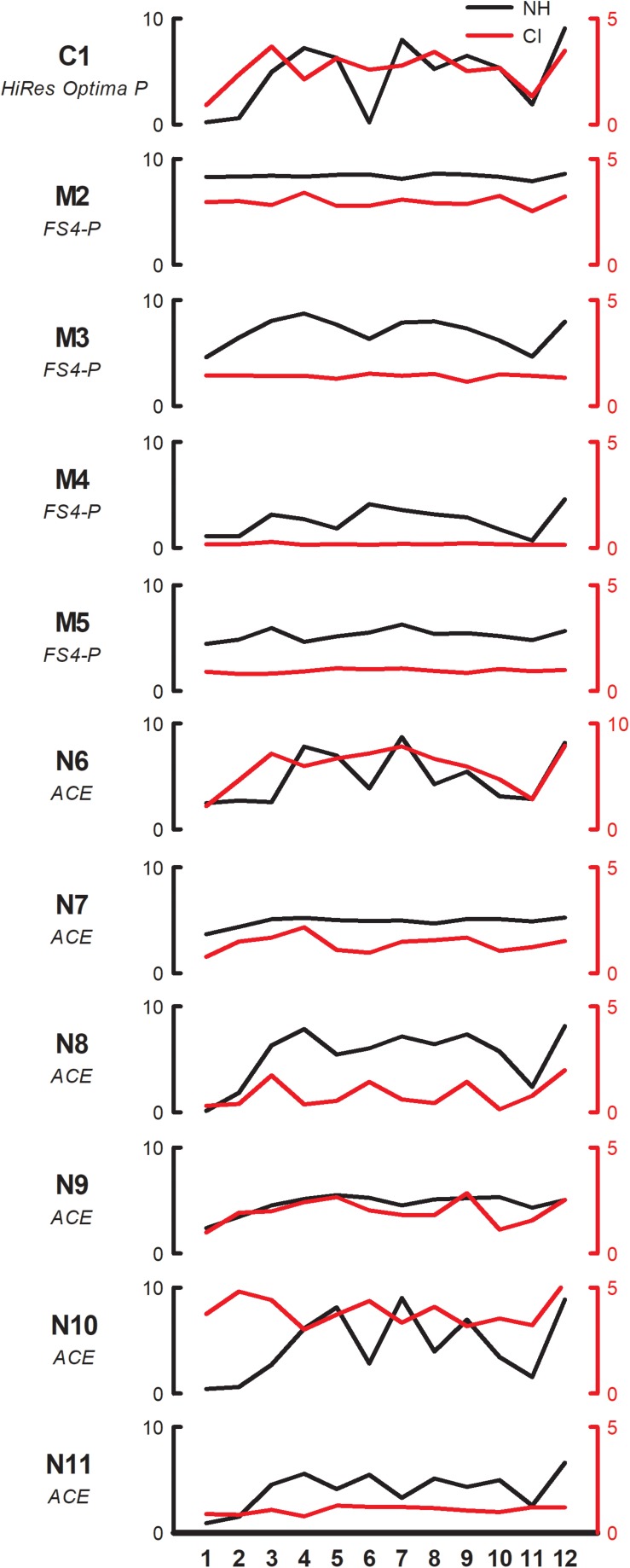
Pleasantness ratings for individual SSD-CI participants with NH-only (black, left ordinate) and CI-only listening (red, right ordinate), for the F3 root note (within-octave) as a function of interval size. Note that the scale for CI-only ratings is half of that for NH-only ratings for all participants except N6. The clinical CI signal processing strategy is listed under each subject’s code.

Figure 2 shows individual pleasantness ratings as a function of interval size for the F3 root note (within-octave interval) with NH-only (black line, left ordinate) and CI-only listening (red line, right ordinate). While the range is much larger with NH-only than with CI-only listening, the rating patterns are somewhat similar between acoustic and electric hearing, especially for some participants (e.g., C1, N8). Most participants rated minor 2nd lowest, whether with acoustic or electric hearing. With NH-only listening, major 3rd, 4th, 5th, and octave were generally rated highest. With CI-only listening, octave was often rated highest; other intervals that also produced relatively high ratings varied across participants. Visual examination of the data showed that some participants exhibited minimal variation in ratings with the CI ear (e.g., M2, M3, M4, M5, N11) while others exhibited minimal variation with the NH ear (M2, N7).

**FIGURE 3:**
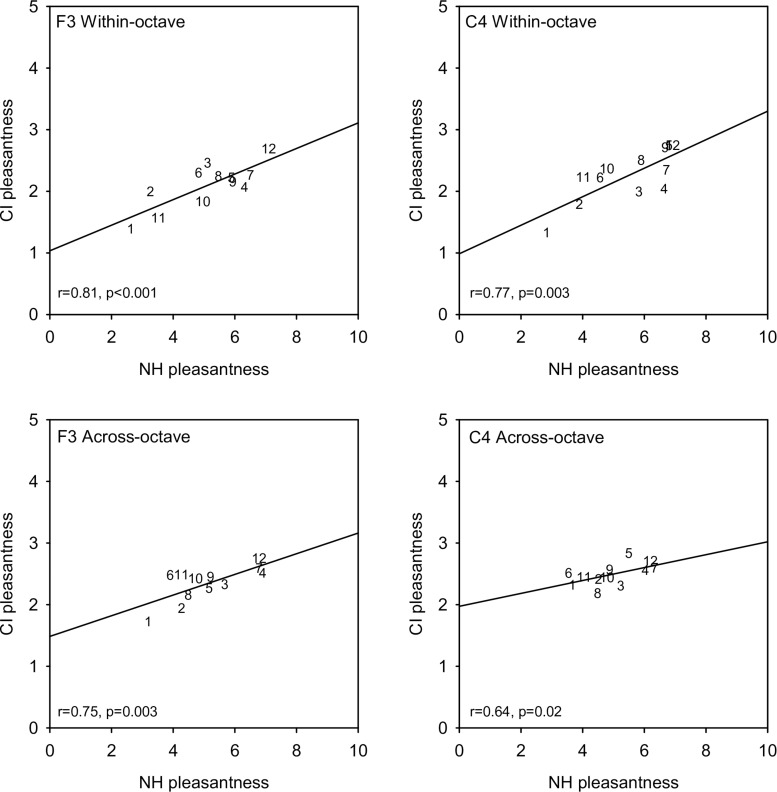
Mean CI-only pleasantness ratings (across subjects) as a function of mean NH-only ratings. Data for the F3 and C4 root notes are shown in the right and left columns, respectively. Data for within- and across-octave intervals are shown in the top and bottom rows, respectively. Note that the range for the CI-only ratings in half of that for the NH-only ratings. Interval size is indicated as numbers in each panel. The diagonal lines show linear regression fits to the data; *r*- and *p*-values are shown for each regression.

Figure 3 shows mean CI-only pleasantness ratings for each interval as a function of NH-only ratings for the different root note and interval span conditions. Linear regressions were fit to the data in each panel; *r*- and *p*-values for each regression are shown at the bottom left in each panel. Strong correlations were observed for all root note and interval span conditions. Across all root notes and interval span conditions, correlations were highly significant between NH-only and CI-only listening (*r* = 0.845, *p* < 0.001), and between NH-only and NH + CI listening (*r* = 0.995, *p* < 0.001). Because of the strong correlations at the group level, additional correlational analyses were performed within individual participants (Supplementary Appendix 3). For NH-only versus CI-only listening, significant correlations (*p* < 0.05) were observed only for participants C1 (F3 within-octave and F3 across-octave), N6 (F3 within-octave and C4 within-octave), and N8 (F3 across-octave). For NH-only versus NH + CI listening, significant correlations (*p* < 0.05) were observed in most cases, with the exceptions of M2 (F3 within-octave, C4 within-octave, C4 across-octave), M5 (F3 within-octave, C4 within-octave, F3 across-octave), and N7 (F3 within-octave). Additional correlational analyses were performed to compare mean ratings across interval span and/or root note for the different listening conditions (Supplementary Appendix 4). For the F3 root note, significant correlations (*p* < 0.01) were observed between within- and across-octave interval ratings for NH-only, CI-only, and NH + CI listening. For C4, significant correlations (*p* < 0.01) were observed between within- and across-octave ratings for NH-only and NH + CI listening, but not for CI-only listening. For both within- and across-octave intervals, significant correlations were observed between F3 and C4 (*p* < 0.015 in all cases). For all root note and interval span conditions, significant correlations were also observed between NH-only and NH + CI listening (*p* < 0.028 in all cases).

### Experiment 1 Discussion

**FIGURE 4:**
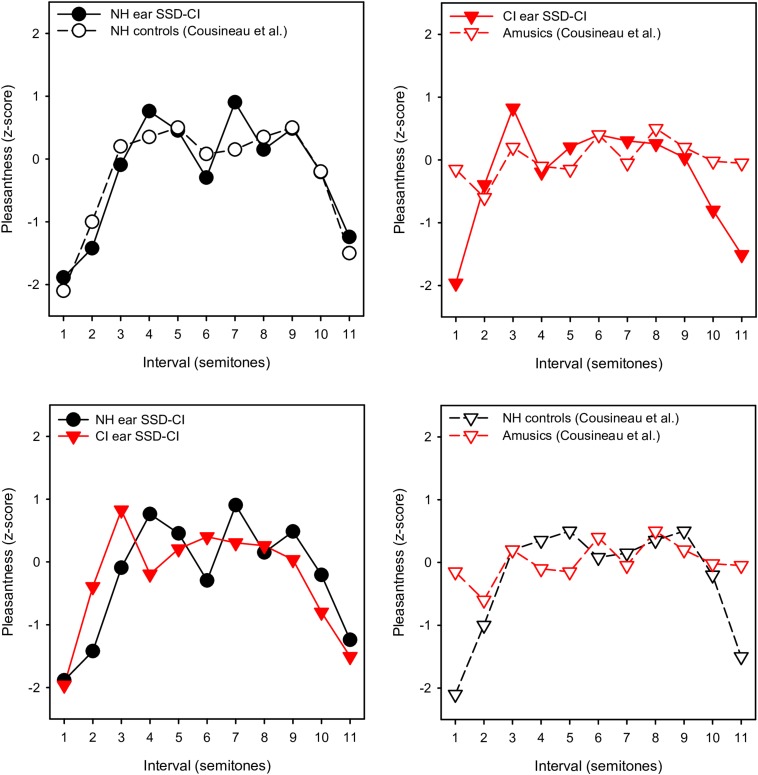
Mean pleasantness ratings (converted to *z*-scores) for SSD-CI participants in the present study (within-octave intervals with F3 root note) and NH controls and amusics from Cousineau et al. (2012). In the **top left panel**, the present SSD-CI NH-only data are plotted alongside the NH control data from Cousineau et al. (2012). In the **top right panel**, the present SSD-CI CI-only data are plotted alongside the amusic data from Cousineau et al. (2012). In the **bottom left panel**, the present SSD-CI NH-only data are plotted alongside the present SSD-CI CI-only data. In the **bottom right panel**, the NH control data from Cousineau et al. (2012) are plotted alongside the amusic data from Cousineau et al. (2012).

With the NH ear, pleasantness ratings were comparable to those for the NH controls in Cousineau et al. (2012). To compare more directly the pattern of results between studies, the present ratings were converted to z-scores, as in Cousineau et al. (2012). Figure 4 shows z-scores for SSD-CI ratings with NH-only and CI-only listening for within-octave intervals with the F3 root note; z-score data for NH controls and amusics were extracted from Figure 2 from Cousineau et al. (2012). Note that the instruments and note ranges used in the experiments differed. NH-only SSD-CI scores were quite similar to the NH control scores from Cousineau et al. (2012). However, CI-only SSD-CI scores were markedly different from amusic scores, especially for minor 2nd and major 7th. Except for major 3rd, tritone, and 5th, SSD-CI z-scores were generally similar between NH-only and CI-only listening. This suggests that beating or roughness may have been a similarly strong cue for both listening conditions. As reported by Cousineau et al. (2012), ratings were markedly different between the NH controls and amusics, especially for minor 2nd and major 7th. Interestingly, the amusics in Cousineau et al. (2012) were able to discriminate between beating and non-beating stimuli, suggesting that the presence of beating may not have driven ratings for minor 2nd and major 7th. The SSD-CI participants in the present study had a normal periphery in one ear, presumably normal central perception of the harmonic intervals (based on the NH-only scores), and a degraded periphery in the other ear. For amusics, the periphery was normal in both ears, discrimination of beating was similar to NH controls, but pleasantness ratings were not consistent with the NH controls, especially for minor 2nd and major 7th, where beating might be a strong cue. Cousineau et al. (2012) suggested that a disordered perception of harmonicity may have given rise to the ambiguous pleasantness ratings in amusics.

As expected, overall ratings were much higher with NH-only than with CI-only listening. Somewhat surprisingly, there was no significant difference between NH + CI and NH-only ratings at the group level. This pattern of results is not consistent with the binaural enhancement observed for the music sound quality ratings (CI-MUSHRA) in SSD-CI participants reported by Landsberger et al. (2019). In that study with SSD-CI listeners, binaural (NH + CI) quality ratings were substantially and significantly higher than with the NH ear alone. Many factors may have contributed to the different patterns of results. First, in Landsberger et al. (2019), two excerpts of musical recordings (“Ring of Fire” and “Rhapsody in Blue”) were used to obtain sound quality ratings. These excerpts were much longer (9–15 s) than the stimuli used in the present study (1 s), and consisted of multiple instruments and/or vocals (versus the piano samples used in the present study). The longer duration and more complex musical sounds may have contributed to the binaural enhancement found in Landsberger et al. (2019). While participants were asked to rate the pleasantness of the stimuli in both studies, participants may have focused attention on different aspects of the stimuli. For the musical intervals presented in the present study, listeners may have attended to the roughness or beating within the stimuli to judge dissonance. The longer duration of the stimuli in Landsberger et al. (2019) may have allowed some qualitative benefit for adding the CI that was not observed in the present study. Note that in Landsberger et al. (2019), the binaural enhancement for SSD-CI users, while substantial, was much less than for NH participants, suggesting that electric hearing was not able to fully restore binaural sound quality for music.

In the present study, binaural ratings for harmonic intervals were clearly dominated by the NH ear, with little contribution from the CI ear. Although one participant (N6) exhibited substantial binaural enhancement, there was no clear factor that appeared to limit binaural enhancement. Previous speech perception studies with bimodal and bilateral CI listeners showed that the degree of binaural enhancement may depend on the degree of performance difference across ears, with binaural enhancement improving as across-ear asymmetry in performance was reduced (Yoon et al., 2011, 2015). Here, there was no significant relationship between the degree of rating asymmetry across ears and the amount of binaural enhancement. It is possible that the general across-ear asymmetry was so large that the relatively minor variability among participants had no effect. In this sense, the present data are consistent with the Yoon et al. studies, in that no binaural enhancement was observed for the highly asymmetrical ratings across ears. Note that in the Yoon et al. studies, speech performance asymmetry was in terms of percent correct; it is unclear whether the pattern of results would have been similar if listeners were asked to rate the quality of speech.

With NH-only or NH + CI listening, the pattern of results for simple intervals was similar to those reported for NH listeners in McDermott et al. (2010) and Cousineau et al. (2012), with minor 2nd, tritone, and major 7th consistently rated lowest, and major 3rd, 4th, 5th, and octave consistently rated highest (top panels of Figure 1). With the CI alone, minor 2nd and major 7th were generally rated lowest and major 3rd, 4th, and octave were generally rated highest. With CI-only listening, there was little sensitivity to the dissonance of the tritone observed with NH-only or NH + CI listening, although some participants (N7, N8, N9, N10) exhibited a dip in ratings in the vicinity of the tritone (red traces in Figure 2). It is possible that due to differences in the electrode-neural interface, the dip in ratings near the tritone may have been slightly shifted across participants. For all three listening conditions, similar rating patterns were observed between within- and across-octave intervals and for both root notes. Statistical analyses showed no significant effects of root note or interval span. The lack of effect of root note suggests differences in frequency allocation were either inconsequential for harmonic interval perception or were varied enough among subjects to wash out any effect of greater dissonance (i.e., temporal beating) for one root note versus the other.

With CI-only listening, participants were likely able to discriminate between an interval presented within or across octaves (e.g., minor 2nd vs. minor 9th) due to differences in the spectral pattern; however, these stimuli were consistently and similarly rated lowest in terms of pleasantness. With CI-only listening, the octave and double octave were generally rated as most pleasant, possibly because of highly periodic temporal envelope cues. However, the place of stimulation for the two component notes did not likely correspond to octave place in the cochlea (Landsberger et al., 2015), given the frequency allocation, electrode neural interface, etc. Thus, while CI users may have perceived the lack of temporal beating in the octave and double octave, they most likely did not receive spectral (harmonicity) cues that would have been available in the NH ear. It is unclear whether the present SSD-CI users perceived octaves similarly between acoustic and electric hearing; however, octaves were most pleasant relative to other intervals with either listening mode.

The lack of effect of interval span for CI listening led us to re-analyze the data using a 3-way RM ANOVA by removing the factor of interval span and considered interval size as a continuous range from 1 to 24. Results were nearly identical to the 4-way RM ANOVA reported above. Therefore, we chose to leave interval span as a factor in order to be consistent with the original study design.

In the present study, harmonic intervals were presented acoustically to the NH ear via headphone and to the CI ear via DAI. For acoustic hearing, listeners could access both temporal envelope interactions between each component interval as well as the degree of “harmonicity” for the combined intervals. For electric hearing, due to the limited spectro-temporal resolution, harmonicity cues would have largely been unavailable; this most likely underlies the overall poor ratings with the CI. As such, temporal envelope interactions (perceived as the degree of roughness or beating in the interval) may have driven differences in ratings across intervals.

**FIGURE 5:**
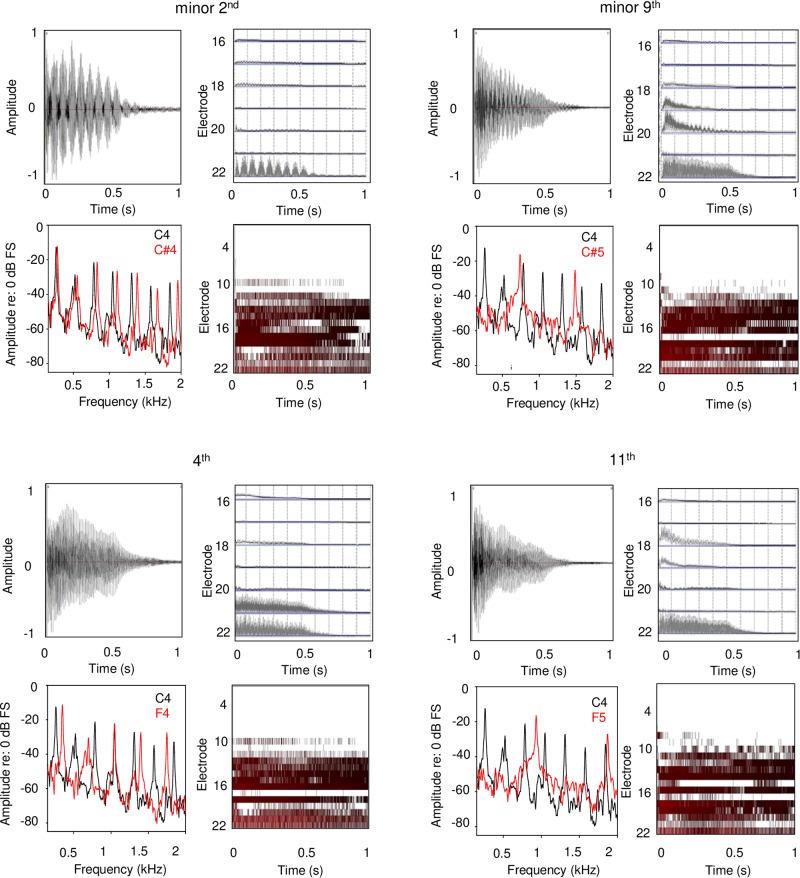
Waveforms, frequency analyses, extracted temporal envelopes, and electrodograms for example dissonant (top panels) and consonant intervals (bottom panels); the left and right panels show within- and across-octave intervals, respectively. The root note for all intervals was C4. Each panel shows (in clockwise order from the top left): waveform, temporal envelopes extracted from each CI signal processor analysis band, electrodogram, and frequency analysis. The extracted temporal envelopes and electrodograms were generated using custom software and using default parameters for Cochlear devices (e.g., ACE strategy, 8 maxima, 900 pulses/second stimulation rate, input frequency range 188–7938 Hz, etc.).

Each panel in Figure 5 shows waveforms and spectra for an acoustic interval, as well as the extracted temporal envelopes for low-frequency channels and electrodograms across all channels for the default CI processor settings for Cochlear devices. For minor 2nd with the C4 root note (top left panel), low-frequency envelope modulation can be observed in the waveform, and the spectra for low-frequency components are slightly offset; these two features would likely give rise to a strong dissonant perception with acoustic hearing. The extracted temporal envelopes exhibit similar beating, especially for the most apical channel (electrode 22). For the electrodogram, there is no clear pattern that would suggest harmonicity or inharmonicity. For minor 9th (top right panel), another typically dissonant interval, the roughness/beating of the temporal envelope can be observed in the waveform, as well as some degree of inharmonicity in the spectra. The roughness/beating can also be observed in the extracted temporal envelope for the CI, especially for electrode 20. For the 4th and 11th intervals (typically rated as consonant), the beating is less apparent in the waveform, and the spectra are more evenly spaced and/or overlapping (greater harmonicity). Overt low-frequency beating is not apparent in electrodograms. While the electrodograms are somewhat different across the minor 2nd, minor 9th, 4th, and 11th intervals, there is no clear pattern that would differentiate any degree of harmonicity among the stimuli. Note that these electrodograms and extracted temporal envelopes specifically apply to Cochlear devices.

Although the temporal and spectral patterns may differ with AB or MED-EL devices, the envelope cues and coarse spectral resolution are likely to be similar for any CI device that represents temporal information by amplitude modulation. In designing the experimental conditions, the different root notes and interval spans were used to accommodate differences among participants’ CI frequency allocations and electrode-neural interfaces (i.e., spectral cues). These accommodations did not appear to affect the general pattern of results, as there were no significant effects of either root note or interval span on CI ratings. For electric hearing, temporal envelope interactions appear to be the dominant cue for the relative dissonance ratings with the CI. This is consistent with Lu et al. (2014), who found that CI users were able to tune a guitar with electric hearing largely by listening to temporal envelope beating. Interestingly, one of the SSD-CI participants in Lu et al. (2014) showed better tuning with acoustic than with electric hearing, possibly due to better access to both harmonicity and temporal beat cues.

To our knowledge, there has been relatively few reports of perception of widely spaced harmonic intervals by NH listeners, outside of early studies by Plomp and Levelt (1965) and Kameoka and Kuriyagawa (1969). These studies suggest greater consonance when notes were separated by an octave or more, possibly because of a greater reliance on spectral than on temporal cues as the notes became more widely separated. Our NH-only data suggest that consonance and dissonance ratings were very similar for the within- and across- octave conditions (e.g., the highly significant correlation between with- and across-octave rating with the NH ear alone in Supplementary Appendix 4, the lack of significant effect of interval span in the RM ANOVA). With CI-only listening, relative dissonance ratings were largely maintained within and across octaves. This suggests that the chroma of the harmonic intervals were largely maintained between acoustic and electric hearing, despite the large differences in sound quality between listening modes.

## Experiment 2: Replication of Ratings for a Reduced Set of Intervals

The data from Exp. 1 showed a significant correlation at the group level for pleasantness ratings between acoustic and electric hearing. However, significant correlations were observed for only a few individual participants. The specific intervals that produced maximum and minimum ratings with the CI may have differed across participants, due to differences in frequency allocation, electrode neural interface, etc. The large number of stimuli tested may have increased the potential for Type 1 error (i.e., finding a significant effect where none exists). It may also have reduced contrasts between strongly consonant and dissonant stimuli. Finally, it was important that the ratings from Exp. 1 be validated for both acoustic and electric hearing to ensure that participants were capable of the task, given that the majority of participants had no musical training. In Exp. 2, pleasantness ratings were obtained for subsets of stimuli that produced the maximum and minimum pleasantness ratings within each listening condition for Exp. 1.

### Methods

#### Participants

The same 11 participants from Exp. 1 also participated in Exp. 2.

#### Stimuli

Test stimuli were generally the same as for Exp. 1, except that only a subset of intervals (i.e., four lowest and four highest rated intervals within each listening condition from Exp. 1) was used for Exp. 2. Note that the lowest and highest four intervals somewhat differed across subjects, especially for CI-only listening. To determine the subset of stimuli for each participant in Exp. 2, the median rating across the five test blocks of Exp. 1 was calculated for each interval within each listening condition. For each participant, ratings were then sorted from low to high within each listening condition; note that stimuli were combined across root note and interval span before sorting. After sorting, the four lowest-rated and the four highest-rated stimuli were identified for each participant for each listening condition from Exp. 1. Thus, for each participant, an optimized stimulus set was created that consisted of the four lowest-rated and the four highest-rated intervals for each listening condition, for a total of 24 stimuli in the stimulus set.

#### Procedures

Test procedures were identical to those in Exp. 1, except that pleasantness ratings were obtained for the stimulus set optimized for each participant and listening condition. As in Exp. 1, five test blocks were administered, and ratings were averaged across the five blocks. Exp. 2 was conducted during the same test session as for Exp. 1, and lasted 30 min. The intervals and listening conditions were randomized within each test block.

### Results

Table 3 shows slopes, *r*- and *p*-values for linear regressions fit between Exp. 1 and Exp. 2 ratings for the optimized stimuli, for each participant and listening condition. Significant positive slopes indicate good replication between Exp. 1 and Exp. 2. Slopes > 1 indicate an expanded range of ratings for Exp. 2 relative to Exp. 1, and slopes < 1 indicate a compressed range of ratings for Exp. 2 relative to Exp. 1. With NH-only listening, significant correlations were observed for all participants (except for M2), with generally high *r*-values (*r* ≥ 0.77). Slopes for these correlations ranged from 0.10 to 1.02. With CI-only listening, significant correlations were observed for all but the MED-EL participants (M2, M3, M4, and M5). Among the significant correlations, *r*-values were generally high (*r* ≥ 0.81). Slopes for these correlations ranged from 0.38 to 1.71. With NH + CI listening, significant correlations were observed for all participants except for M2. Slopes ranged from 0.29 to 1.37. The high number of significant correlations suggest that for most participants, the results from Exp. 1 were replicated in Exp. 2. Note that the slopes for most of the correlation functions were < 1, indicating that the range of ratings for Exp. 2 were compressed relative to Exp. 1.

**TABLE 3:** Results of linear regressions between Exp. 1 and Exp. 2 rating data.

	**NH-only**			**CI-only**			**NH + CI**		
			
**Subject**	**Slope**	***r***	***p***	**Slope**	***r***	***p***	**Slope**	***r***	***p***
C1	0.98	0.99	< 0.001^∗^	1.71	0.93	< 0.001^∗^	0.96	0.99	< 0.001^∗^
M2	0.01	0.05	0.899	0.36	0.16	0.708	0.04	0.42	0.303
M3	0.81	0.95	< 0.001^∗^	0.15	0.57	0.137	0.62	0.97	< 0.001^∗^
M4	0.70	0.84	0.008^∗^	–0.02	0.07	0.864	1.03	0.97	< 0.001^∗^
M5	1.02	0.96	< 0.001^∗^	–0.09	0.22	0.595	0.90	0.83	0.011^∗^
N6	0.97	0.98	< 0.001^∗^	0.97	0.99	< 0.001^∗^	1.05	0.99	< 0.001^∗^
N7	0.10	0.77	0.025^∗^	0.57	0.81	0.014^∗^	0.29	0.92	0.001^∗^
N8	0.86	0.99	< 0.001^∗^	0.71	0.84	0.010^∗^	0.92	0.99	< 0.001^∗^
N9	0.97	0.92	0.001^∗^	0.56	0.88	0.004^∗^	1.37	0.85	0.007^∗^
N10	0.95	0.99	< 0.001^∗^	0.56	0.86	0.006^∗^	0.99	0.99	< 0.001^∗^
N11	0.85	0.99	< 0.001^∗^	0.38	0.91	0.002^∗^	0.86	0.98	< 0.001^∗^

*Pleasantness ratings for the four lowest rated and four highest rated stimuli (across root notes and interval spans) in Exp. 1 were re-measured in Exp. 2. Linear regressions were fit to these data for each participant. The asterisks indicate significant correlations between Exp. 1 and Exp. 2 data. The shaded cells indicate instances where participants were unable to replicate the data from Exp. 1.*

### Experiment 2 Discussion

Given the significant correlations between Exp. 1 and Exp. 2 for NH-only listening, the replication data suggest that 10 of the 11 participants were able to reliably rate relative dissonance with the present stimuli and methods. The same pattern of results was found for NH + CI listening. It is unclear why participant M2 was unable to replicate the data from Exp. 1. For M2, the range of ratings (across all stimuli) was 7.9–8.7; for the remaining participants, the range was 1.9–4.5. It is unclear why there was so little differentiation in NH-only ratings for M2. It is possible that all intervals sounded pleasant in general, or that the typical distinctions between consonant and dissonant stimuli were not perceived as different.

Too few patients were tested to compare results across devices fairly. Nevertheless, it seems noteworthy that while AB and Cochlear users were able to replicate the CI ratings from Exp. 1, none of the MED-EL users were able to do so. Excluding M2 (who was unable to reliably perform the task with NH-only listening), the range of ratings (across all stimuli) with CI-only listening for the remaining three MED-EL participants was 0.1–1.9, and the mean rating was 0.9. For the AB and Cochlear participants, the range of ratings was 0.1–8.6, and the mean rating was 2.8. Thus, the three MED-EL participants exhibited a smaller range and lower mean ratings than did the AB and Cochlear participants. It is possible that the differences in ratings among the devices may be due to differences in encoding temporal information across CI signal processing strategies. The AB and Cochlear participants used Optima and ACE processing, respectively. These strategies represent temporal information extracted from frequency analysis channels by modulating fixed-rate pulse trains delivered to the appropriate electrodes. MED-EL’s FS4-p processing similarly modulates fixed-rate pulse trains delivered to electrodes 5–12 (which typically represent 710 – 8500 Hz). However, on the most apical electrodes (1–4, which typically represent 100–710 Hz), modulation is applied to variable rate pulse trains that correspond to the zero-crossings in each channel. As such, the F0s for the component notes for most intervals would have been delivered to “fine-structure” (FS) channels 1–4; harmonic information above 710 Hz would have been delivered to the fixed-rate channels 5–12. With the FS channels, frequency is primarily encoded by rate of stimulation and temporal beating would be encoded by a temporal jitter rather than amplitude modulation (AM) beating. It is therefore possible that perception of temporal beating (especially for minor 2nd and major 7th) may have differed for FS4-P and the envelope-based strategies. Again, this pattern of results may have been due to the interaction between the relatively low root notes and the frequency allocation for the FS channels in MED-EL users. With higher root notes (e.g., A5, or 880 Hz), the fixed-rate channels 5–12 would have been stimulated and the pattern of results may have been more comparable to those of the present AB and Cochlear users.

For most participants, the slopes between Exp. 1 and Exp. 2 ratings were close to 1 for NH-only and NH + CI listening, suggesting that ratings for these stimuli were largely the same when presented as a subset of the larger group tested in Exp. 1. For most of the participants who replicated the Exp. 1 data with CI-only listening, slopes were considerably less than 1 suggesting that with the reduced stimulus set in Exp. 2 produced a smaller range of ratings. This suggests that with the smaller stimulus set in Exp. 2, relative dissonance was less pronounced than with the larger stimulus set from Exp. 1.

## General Discussion

The SSD-CI participants tested in the present study represent a unique patient population with which to explore perception of harmonic intervals. Pleasantness ratings with the NH ear provided an estimate of participants’ abilities to perceive relative dissonance; ratings were similar to those for NH listeners in previous studies (e.g., McDermott et al., 2010; Cousineau et al., 2012). Via DAI, ratings were also obtained with CI-only listening. With combined acoustic and electric hearing, the relative contributions of the NH and CI ears could be observed. Thus, the data set with SSD-CI listeners provides direct comparison of pleasantness rating for very different peripheral representations within the same participant. The generally low CI-only ratings are consistent with previous studies that show difficulties in CI users’ perception of polyphonic music (e.g., Donnelly et al., 2009; Galvin et al., 2009b, 2012; Penninger et al., 2013, 2014; Crew et al., 2015; Chari et al., 2019). The present pattern of results with CI-only listening is also generally consistent with Knobloch et al. (2018), who found that adult post-lingually deaf CI users rated major chords as sounding more pleasant than minor augmented, suspended, and diminished chords. Similar to previous studies with bimodal CI listeners (e.g., Crew et al., 2015, 2016), the NH ear appeared to drive binaural quality ratings, with the CI ear contributing little to binaural ratings.

Despite the large difference in absolute ratings between NH-only and CI-only listening (Figure 1), perception of relative dissonance was largely similar between acoustic and electric hearing (Figures 1–3). This suggests that while CI-only ratings were generally poor, some intervals consistently sounded less pleasant than others. This finding was not fully in agreement with our hypothesis that CI-only ratings would be poor, with little relation to the pattern of ratings with NH-only listening. The CI-only data suggest that participants were mostly sensitive to dissonance (roughness, perceived for minor 2nd, major 7th, and minor 9th), with some sensitivity to consonance (“harmonicity,” perceived for the octave); for the remaining intervals, pleasantness ratings were largely similar. The transitions in ratings from minor 2nd to major 3rd and from major 6th to major 7th were generally similar between acoustic and electric hearing (at least for the F3 root note). Ratings were highly correlated across root notes and interval spans (Supplementary Appendix 4), suggesting that the spectral pattern did not contribute strongly to relative dissonance ratings. The electrodograms in Figure 5 also do not indicate any useful distinctions that might underlie ratings. Given that differences in spectral patterns did not seem to contribute to CI-only ratings, it is likely that temporal envelope cues were responsible for the relative dissonance ratings in Exp. 1. Note that 3 of the 10 participants who were able to replicate Exp. 1 ratings in Exp. 2 with NH-only listening were unable to do so with CI-only listening. As discussed previously, these participants were all MED-EL users, and interactions between the root notes tested and the FS4-p processing may have contributed to the pattern of results. If so, this would suggest that it is important to accurately preserve temporal envelope cues across all channels to perceive dissonance with electric hearing.

Interestingly, the present CI-only data suggest that while stimuli sounded generally inharmonic (due to poor spectral resolution), relative pleasantness ratings hewed close to NH-only ratings. This suggests that beating, rather than perception of inharmonicity played a stronger role in relative ratings with CI-only listening. The similarity between the NH-only and CI-only data further suggest that temporal information (e.g., beating) may play a strong role in dissonance perception, and that the degree of underlying harmonicity may not affect contrasts between consonant and dissonant intervals. This is not to say that harmonicity is not important when rating the pleasantness of a sound. Clearly, CI-only ratings were generally poor, and were lower than the least-pleasant NH-only ratings (Figure 1). However, when data were compared across listening modes (Figure 3) the pattern of ratings was quite similar, despite the large differences in spectral resolution across ears.

Several studies have shown that CI users are susceptible to temporal modulation interference, even when temporal envelope information is spatially distant (e.g., Chatterjee, 2003; Kreft et al., 2013; Chatterjee and Kulkarni, 2018). In such modulation detection interference (MDI) studies, CI users were asked to detect an amplitude-modulated (AM) probe stimulus in the presence of masking AM stimuli with similar or different AM rates as the probe; the electrode positions of the masker and probe stimuli were varied to be spatially proximate (which would produce the most energetic masking and perhaps the greatest interference) or spatially remote (which would produce less energetic masking and perhaps less interference). After accounting for energetic masking, significant amounts of “modulation masking” were observed even when electrodes were spatially remote. Modulation masking may have been due to the broad current spread associated with electric stimulation and/or to more central processing of temporal envelope information (e.g., Dau et al., 1997). In MDI studies, masker and probe AM rates are typically selected to avoid low-frequency beating and/or harmonically related AM rates.

In the present study, the harmonic intervals would be expected to produce different degrees of beating and harmonically related temporally envelope information. These musical intervals are used to compose Western music and are therefore commonly experienced. Different from MDI studies that suggest that modulation masking may limit perception of important temporal envelope cues, the present data suggest that CI users may use temporal envelope interaction cues to make qualitative judgments about a stimulus in ways that are similar to acoustic hearing. While the underlying mechanisms may be similar between MDI and harmonic interval perception, the listening task may give rise to different percepts (detection of a masked AM stimulus versus rating the pleasantness of combined temporal envelope information). Although the across-octave intervals in the present study did not offer the same degree of electrode separation as in some MDI CI studies, pleasantness ratings were very similar for within- and across-octave intervals. It is unclear whether this was due to the broad current spread associated with electric stimulation or to central processing of temporal envelope cues. As shown in Figure 5, the temporal envelope information associated with dissonant intervals (due to beating) was largely preserved by CI signal processing. One advantage in CI research is the ability to separate temporal (AM, stimulation rate) and spectral cues (electrode location), which has been exploited in MDI studies. In future CI research, it would be worthwhile to study how dissonance ratings are affected by both temporal envelope interactions and electrode interactions. Finally, the generally low CI ratings may be due in part to temporal envelope interactions across all electrodes. The MDI data from previous studies suggest strong temporal envelope interactions even when electrodes are spatially separated. For the present harmonic intervals, such widespread temporal envelope interactions may reduce overall sound quality.

Contrary to our hypothesis, we did not find significant binaural enhancement for interval ratings for the present SSD-CI listeners. This finding is also not consistent with MUSHRA ratings from SSD-CI listeners in Landsberger et al. (2019) or from SSD-CI questionnaire data that suggest better overall sound quality for binaural listening after cochlear implantation (e.g., Távora-Vieira et al., 2013; Friedmann et al., 2016; Dillon et al., 2017b; Galvin et al., 2018). The short stimulus duration and other methodological factors may have limited binaural enhancement in the present study. Longer musical excerpts (as used in Landsberger et al., 2019) may be necessary to perceive a qualitative binaural advantage over NH-only listening. Similarly, longer-term experience with binaural listening (along with SSD-CI patients’ anecdotal reports) may give rise to a stronger sense of binaural enhancement. It is worth noting that in many of these SSD-CI studies, there are typically small benefits for binaural speech understanding when speech and noise are co-located, despite subjective data that suggest strong binaural enhancement. Indeed, SSD-CI users are often surprised by the poor sound quality and intelligibility with CI-only compared to NH + CI listening. The NH ear seems to capture the quality of binaural listening somehow, despite the likely asymmetry in quality across ears. As such, the source of binaural enhancement is unclear. It may be that some gross binaural restoration improves sound quality over monaural, NH-only listening. However, these potentially strong top-down effects may not provide useful information toward improving CI sound quality, which is important to improve binaural sound quality. The present harmonic interval perception data may provide insights into the limited contribution of the CI to combined acoustic-electric sound quality. Ideally, both absolute and relative dissonance patterns observed with the NH ear should be preserved with the CI ear. Understanding the limits of the CI ear may guide future CI signal processing and technology to improve CI quality for music perception, thereby improving binaural enhancement for SSD-CI (and possibly, bimodal and bilateral CI) patients.

Note that only pleasantness ratings were measured in the present study. It is unclear how discriminable these intervals were, especially with the CI ear alone. The relationship between interval discrimination and pleasantness is unclear. With the NH ear alone, it is likely that all intervals would be reliably discriminated, but some intervals would have been similarly rated (e.g., major 3rd, 5th, major 10th, 12th). With the CI ear alone, it is unclear how discriminable some intervals were. For example, the dip in ratings for the tritone with the NH ear alone was not observed with the CI ear alone. It is possible that CI users may not have been able to discriminate among the 4th, tritone, and 5th. However, they are likely to have been able to discriminate between a minor 2nd and a minor 9th on the basis of the spectral patterns; yet these stimuli were rated similarly unpleasant for the F3 root note. In future studies, it may be useful to measure discrimination as well as similarity ratings (using multi-dimensional scaling) for harmonic intervals with acoustic and electric hearing.

Testing with SSD-CI users allowed us to verify whether the pattern of dissonance ratings observed with CI-only listening was related to the pattern with the NH ear (which was presumably and decidedly similar to that of NH listeners in general). The strong correlations between the NH-only and CI-only ratings, as well as the replication of ratings in Exp. 2 further support the relationship between acoustic and electric hearing for relative dissonance ratings for harmonic intervals. However, it is important to note that SSD-CI users most likely do not rely on their implant in the same way as bilaterally deaf CI users, which may influence their perception of sound through the device and in this case, the dissonance of harmonic intervals. Therefore, while using the SSD-CI population allowed for assurance of task understanding, especially in a group with very little formal musical training, it may be difficult to generalize these results to bilaterally or asymmetrically deaf CI users. In particular, considering that very few participants showed correlations between ratings in the NH-only and CI-only conditions, it may not be expected that each individual has access to the perception of dissonance through the CI.

The present study sheds new light on CI users’ difficulty with consonance perception for harmonic intervals. Many previous studies have shown that CI users have great difficulty with melodic interval perception (e.g., Gfeller and Lansing, 1991; Gfeller et al., 2002, 2007; Galvin et al., 2007, 2009a; Looi et al., 2008; Nimmons et al., 2008). Distortions to interval size (due to frequency allocation, the limited number of electrodes, and the electrode-neural interface) result in distortions to melody. While factors that underlie poor melodic interval perception may have contributed to the overall poor ratings in harmonic interval perception, the present results suggest that some aspects of harmonic interval perception observed with acoustic hearing were preserved with electric hearing. Note that the present methodology was restricted to acute pleasantness ratings for isolated “vertical” intervals. In ongoing polyphonic music, there are horizontal and vertical dimensions that can be analyzed independently and synthesized together. Although CI users may be able to analyze some aspects of harmony, they have difficulty synthesizing harmony and melody. In Knobloch et al. (2018), CI users’ chord preferences were generally similar to those of NH listeners. Unlike NH listeners, CI users were unable to perceive “authentic cadences” at the end of a musical excerpt, indicating difficulties in perceiving harmonic “syntax.” Thus, relative dissonance perception was not helpful in perceiving the resolution of a polyphonic melody. Given the limits of current CI technology, it may not be possible to achieve the necessary spectral resolution to support good polyphonic music perception. Optimizing combined acoustic and electric hearing (e.g., reducing interaural frequency mismatch, reducing CI channel interaction, etc.) may be the most promising approach toward improving music perception in CI users.

## Conclusion

In the present study, pleasantness ratings for harmonic intervals were measured in adult, unilaterally deaf CI users while listening with the CI ear alone, the NH ear alone, and with both ears (NH + CI). The root note and interval span (within-or across-octave) were varied to accommodate differences across participants in terms of the acoustic-to-electric frequency allocation and electrode-neural interface. Findings include:

1. Overall ratings were much poorer with the CI than with NH. There was no significant binaural enhancement with NH + CI over NH-only listening.
2. Despite the large asymmetry in overall ratings across ears, significant correlations were observed between NH-only and CI-only ratings, suggesting that relative dissonance was similar between acoustic and electric hearing.
3. All but one of the SSD-CI participants were able to replicate relatively low and high ratings with NH-only listening with a smaller, high-contrast stimulus set. Only some participants were able to replicate ratings with CI-only listening.
4. There was no significant effect of root note or interval span on ratings for any of the listening conditions, suggesting that spectral cues did not contribute strongly to CI-only interval ratings. More likely, temporal envelope interactions (beating, roughness) contributed to relative dissonance perception with electric hearing.

## Data Availability

The datasets generated for this study are available on request to the corresponding author.

## Ethics Statement

All participants were paid for their participation and provided written informed consent in accordance with the study protocols reviewed and approved by Institutional Review Board at New York University (IRB #S14-00809 and #S14-00435).

## Author Contributions

ES, DL, and JG contributed to the study design, creation of stimuli, statistical analyses, and writing of the manuscript. ES contributed to the participant recruitment and data collection. DL wrote the software used to run the experiments and collect the data. DF contributed to the study design and writing of the manuscript. All authors read and approved the final submitted version.

## Conflict of Interest Statement

The authors declare that the research was conducted in the absence of any commercial or financial relationships that could be construed as a potential conflict of interest. The handling Editor JM declared a past co-authorship with one of the authors, DL.

## Footnotes

1 http://kenschutte.com/midi
