# Supplementary material for: Pleasantness Ratings for Harmonic Intervals With Acoustic and Electric Hearing in Unilaterally Deaf Cochlear Implant Patients
Source: Front Neurosci. 2019 Sep 3;13:922. doi: 10.3389/fnins.2019.00922 (PMC6733976; doi:10.3389/fnins.2019.00922)
Supplement: Supplementary file 1 [file Table_1.DOCX]

|  | **NH-only, CI-only, NH+CI** | | | **NH-only, NH+CI** | | | **NH-only, CI-only** | | | **CI-only, NH+CI** | | |
| --- | --- | --- | --- | --- | --- | --- | --- | --- | --- | --- | --- | --- |
|  | **dF, res** | **F** | **p** | **dF, res** | **F** | **p** | **dF, res** | **F** | **p** | **dF, res** | **F** | **p** |
| Interval | 11, 110 | 14.95 | <0.001* | 11, 110 | 14.26 | <0.001* | 11, 110 | 13.53 | <0.001* | 11, 110 | 15.49 | <0.001* |
| Interval span | 1, 10 | 0.20 | 0.662 | 1, 10 | 1.08 | 0.323 | 1, 10 | 0.03 | 0.867 | 1, 10 | 0.51 | 0.491 |
| Root note | 1, 10 | 4.25 | 0.066 | 1, 10 | 1.78 | 0.211 | 1, 10 | 4.72 | 0.055 | 1, 10 | 8.37 | 0.016* |
| Ear | 2, 20 | 18.12 | <0.001* | 1, 10 | 0.83 | 0.383 | 1, 10 | 20.48 | 0.001* | 1, 10 | 20.26 | 0.001* |
| Interval * Interval span | 11, 110 | 10.27 | <0.001* | 11, 110 | 9.55 | <0.001* | 11, 110 | 8.04 | <0.001* | 11, 110 | 8.30 | <0.001* |
| Interval *  Root note | 11, 110 | 5.20 | <0.001* | 11, 110 | 3.55 | <0.001* | 11, 110 | 4.09 | <0.001* | 11, 110 | 4.94 | <0.001* |
| Interval span *  Root note | 1, 10 | 6.94 | 0.025* | 1, 10 | 7.29 | 0.022* | 1, 10 | 5.61 | 0.039* | 1, 10 | 6.26 | 0.031* |
| Interval *  Ear | 2, 220 | 9.29 | <0.001* | 11, 110 | 0.93 | 0.511 | 11, 110 | 9.05 | <0.001* | 11, 110 | 11.17 | <0.001* |
| Interval span * Ear | 2, 20 | 3.60 | 0.046* | 1, 10 | 6.44 | 0.029 | 1, 10 | 4.20 | 0.068 | 1, 10 | 2.69 | 0.132 |
| Root note * Ear | 2, 20 | 0.61 | 0.552 | 1, 10 | 4.36 | 0.063 | 1, 10 | 0.23 | 0.643 | 1, 10 | 0.22 | 0.648 |
| Interval * Interval span * Root note | 11, 110 | 1.87 | 0.049* | 11, 110 | 1.60 | 0.109 | 11, 110 | 2.25 | 0.016* | 11, 110 | 1.85 | 0.054 |
| Interval * Interval span * Ear | 2, 220 | 2.75 | <0.001* | 11, 110 | 0.55 | 0.866 | 11, 110 | 3.50 | <0.001* | 11, 110 | 3.07 | 0.001* |
| Interval *  Root note * Ear | 2, 220 | 0.86 | 0.651 | 11, 110 | 0.67 | 0.766 | 11, 110 | 0.92 | 0.521 | 11, 110 | 0.99 | 0.461 |
| Interval span *  Root note * Ear | 2, 20 | 5.41 | 0.013* | 1, 10 | 0.67 | 0.431 | 1, 10 | 6.08 | 0.033* | 1, 10 | 7.37 | 0.022* |
| Interval * Interval span * Root note * Ear | 2, 220 | 1.62 | 0.043* | 11, 110 | 1.40 | 0.185 | 11, 110 | 1.66 | 0.093 | 11, 110 | 1.74 | 0.073 |

Appendix 1. Results of multi-way RM ANOVAs for Exp. 1 data. The asterisks indicate significant effects.
