# Supplementary material for: Pleasantness Ratings for Harmonic Intervals With Acoustic and Electric Hearing in Unilaterally Deaf Cochlear Implant Patients
Source: Front Neurosci. 2019 Sep 3;13:922. doi: 10.3389/fnins.2019.00922 (PMC6733976; doi:10.3389/fnins.2019.00922)
Supplement: Supplementary file 2 [file Table_2.DOCX]

| **Participant** | **t (dF=47)** | **p** |  |
| --- | --- | --- | --- |
| C1 | 2.5 | 0.015* | NH-only>NH+CI |
| M2 | 13.9 | <0.001* | NH-only>NH+CI |
| M3 | -0.3 | 0.791 |  |
| M4 | -1.3 | 0.207 |  |
| M5 | 1.4 | 0.157 |  |
| N6 | <0.1 | 0.996 |  |
| N7 | -31.0 | <0.001* | NH+CI>NH-only |
| N8 | 1.1 | 0.288 |  |
| N9 | -19.5 | <0.001* | NH+CI>NH-only |
| N10 | 1.2 | 0.232 |  |
| N11 | 1.3 | 0.213 |  |

Appendix 2. Results of t-tests performed on interval rating data for each participant from Exp. 1 comparing NH-only and NH+CI performance; data were pooled across interval span and root note conditions. The asterisks indicate significant effects.
