# Supplementary material for: Pleasantness Ratings for Harmonic Intervals With Acoustic and Electric Hearing in Unilaterally Deaf Cochlear Implant Patients
Source: Front Neurosci. 2019 Sep 3;13:922. doi: 10.3389/fnins.2019.00922 (PMC6733976; doi:10.3389/fnins.2019.00922)
Supplement: Supplementary file 3 [file Table_3.DOCX]

|  |  |  | **NH-only vs CI-only** | | | | **NH-only vs NH+CI** | | | |
| --- | --- | --- | --- | --- | --- | --- | --- | --- | --- | --- |
|  |  | **Participant** | **Slope** | **r** | **p** | **Slope** | | **r** | **p** |  |
| Within-Octave | F3 | C1 | 0.17 | 0.64 | 0.018* | 0.77 | | 0.85 | <0.001* |  |
|  |  | M2 | 0.36 | 0.25 | 0.418 | 0.09 | | 0.05 | 0.883 |  |
|  |  | M3 | -0.02 | 0.24 | 0.432 | 1.04 | | 0.89 | <0.001* |  |
|  |  | M4 | 0.02 | 0.39 | 0.194 | 0.37 | | 0.60 | 0.029* |  |
|  |  | M5 | 0.04 | 0.22 | 0.476 | 0.52 | | 0.54 | 0.058 |  |
|  |  | N6 | 0.53 | 0.66 | 0.014* | 1.02 | | 0.94 | <0.001* |  |
|  |  | N7 | 0.25 | 0.36 | 0.232 | 0.99 | | 0.53 | 0.061 |  |
|  |  | N8 | 0.16 | 0.55 | 0.051 | 0.95 | | 0.93 | <0.001* |  |
|  |  | N9 | 0.32 | 0.49 | 0.088 | 0.60 | | 0.65 | 0.017* |  |
|  |  | N10 | -0.03 | 0.15 | 0.622 | 0.98 | | 0.98 | <0.001* |  |
|  |  | N11 | 0.05 | 0.38 | 0.202 | 0.76 | | 0.89 | <0.001* |  |
|  |  | AVG | 0.22 | 0.81 | <0.001* | 1.02 | | 0.98 | <0.001* |  |
|  | C4 | C1 | 0.12 | 0.49 | 0.087 | 0.91 | | 0.99 | <0.001* |  |
|  |  | M2 | -0.33 | 0.23 | 0.451 | -0.98 | | 0.41 | 0.170 |  |
|  |  | M3 | 0.02 | 0.25 | 0.413 | 0.98 | | 0.92 | <0.001* |  |
|  |  | M4 | 0.02 | 0.28 | 0.361 | 0.80 | | 0.73 | 0.004* |  |
|  |  | M5 | -0.01 | 0.06 | 0.841 | 0.24 | | 0.44 | 0.135 |  |
|  |  | N6 | 0.44 | 0.73 | 0.005* | 1.03 | | 0.97 | <0.001* |  |
|  |  | N7 | 0.34 | 0.40 | 0.181 | 1.04 | | 0.67 | 0.013* |  |
|  |  | N8 | 0.13 | 0.44 | 0.136 | 0.88 | | 0.95 | <0.001* |  |
|  |  | N9 | 0.37 | 0.40 | 0.180 | 0.84 | | 0.79 | <0.001* |  |
|  |  | N10 | 0.12 | 0.37 | 0.217 | 0.79 | | 0.91 | <0.001* |  |
|  |  | N11 | 0.01 | 0.01 | 0.996 | 0.75 | | 0.89 | <0.001* |  |
|  |  | AVG | 0.23 | 0.77 | 0.002* | 0.97 | | 0.99 | <0.001* |  |
| Across-Octave | F3 | C1 | 0.14 | 0.60 | 0.030* | 0.94 | | 0.95 | <0.001* |  |
|  |  | M2 | -1.18 | 0.36 | 0.231 | 1.87 | | 0.80 | 0.001* |  |
|  |  | M3 | 0.02 | 0.17 | 0.571 | 0.96 | | 0.86 | <0.001* |  |
|  |  | M4 | 0.02 | 0.12 | 0.693 | 0.55 | | 0.65 | 0.017* |  |
|  |  | M5 | 0.02 | 0.14 | 0.657 | 0.21 | | 0.26 | 0.383 |  |
|  |  | N6 | 0.25 | 0.54 | 0.056 | 0.96 | | 0.98 | <0.001* |  |
|  |  | N7 | 0.88 | 0.50 | 0.079 | 2.08 | | 0.71 | 0.006* |  |
|  |  | N8 | 0.11 | 0.62 | 0.024* | 0.94 | | 0.96 | <0.001* |  |
|  |  | N9 | 0.26 | 0.37 | 0.208 | 0.71 | | 0.74 | 0.004* |  |
|  |  | N10 | 0.07 | 0.20 | 0.523 | 0.88 | | 0.86 | <0.001* |  |
|  |  | N11 | 0.05 | 0.26 | 0.389 | 0.75 | | 0.82 | <0.001* |  |
|  |  | AVG | 0.17 | 0.75 | 0.003* | 1.02 | | 0.99 | <0.001* |  |
|  | C4 | C1 | -0.01 | 0.07 | 0.827 | 0.96 | | 0.94 | <0.001* |  |
|  |  | M2 | 0.06 | 0.05 | 0.864 | -0.28 | | 0.13 | 0.679 |  |
|  |  | M3 | 0.03 | 0.26 | 0.391 | 0.85 | | 0.85 | <0.001* |  |
|  |  | M4 | -0.04 | 0.26 | 0.393 | 0.90 | | 0.75 | 0.003* |  |
|  |  | M5 | 0.09 | 0.36 | 0.232 | 0.72 | | 0.74 | 0.004* |  |
|  |  | N6 | 0.16 | 0.37 | 0.220 | 1.23 | | 0.98 | <0.001* |  |
|  |  | N7 | 0.13 | 0.09 | 0.779 | 1.72 | | 0.69 | 0.009* |  |
|  |  | N8 | 0.06 | 0.23 | 0.452 | 0.72 | | 0.70 | 0.008* |  |
|  |  | N9 | 0.32 | 0.41 | 0.161 | 0.72 | | 0.62 | 0.023* |  |
|  |  | N10 | 0.15 | 0.51 | 0.075 | 0.71 | | 0.91 | <0.001* |  |
|  |  | N11 | 0.01 | 0.03 | 0.923 | 0.64 | | 0.88 | <0.001* |  |
|  |  | C1 | 0.11 | 0.64 | 0.020* | 1.02 | | 0.98 | <0.001* |  |
| Grand Average |  | AVG | 0.19 | 0.85 | <0.001* | 1.02 | | 0.99 | <0.001* |  |

Appendix 3. Results of linear regression analyses comparing NH-only vs. CI-only and NH-only vs. NH+CI ratings within participants for the different root note and interval span conditions. Asterisks indicate significant correlations.
