# Supplementary material for: Pleasantness Ratings for Harmonic Intervals With Acoustic and Electric Hearing in Unilaterally Deaf Cochlear Implant Patients
Source: Front Neurosci. 2019 Sep 3;13:922. doi: 10.3389/fnins.2019.00922 (PMC6733976; doi:10.3389/fnins.2019.00922)
Supplement: Supplementary file 4 [file Table_4.DOCX]

|  |  |  | **Slope** | **r** | **p** |
| --- | --- | --- | --- | --- | --- |
| NH-only vs CI-only | Within | F3 | 0.22 | 0.81 | <0.001* |
|  |  | C4 | 0.23 | 0.77 | 0.002* |
|  | Across | F3 | 0.17 | 0.75 | 0.003* |
|  |  | C4 | 0.11 | 0.64 | 0.020* |
| NH-only vs NH+CI | Within | F3 | 1.02 | 0.98 | <0.001* |
|  |  | C4 | 0.97 | 0.99 | <0.001* |
|  | Across | F3 | 1.02 | 0.99 | <0.001* |
|  |  | C4 | 1.02 | 0.98 | <0.001* |
| Within- vs. Across-Octave | NH-only | F3 | 0.94 | 0.87 | <0.001* |
|  |  | C4 | 1.01 | 0.79 | 0.001* |
|  | CI-only | F3 | 0.83 | 0.63 | 0.021* |
|  |  | C4 | 1.23 | 0.56 | 0.045* |
|  | NH+CI | F3 | 0.97 | 0.88 | <0.001* |
|  |  | C4 | 0.99 | 0.83 | <0.001* |
| F3 vs C4 | NH-only | within | 0.98 | 0.95 | <0.001* |
|  |  | across | 1.17 | 0.96 | <0.001* |
|  | CI-only | within | 0.57 | 0.60 | 0.031* |
|  |  | across | 0.92 | 0.59 | 0.034* |
|  | NH+CI | within | 1.06 | 0.97 | <0.001* |
|  |  | across | 1.14 | 0.95 | <0.001* |

Appendix 4. Results of linear regressions comparing mean ratings across listening conditions, interval spans, and root notes. Asterisks indicate significant correlations.
